# Correction to “Safety Considerations and Proposed Workflow for Laboratory Scale Chemical Synthesis by Ball Milling”

**DOI:** 10.1021/acs.oprd.3c00155

**Published:** 2023-06-07

**Authors:** Ian Priestley, Claudio Battilocchio, Andrei Iosub, Fabien Barreteau, Gavin W. Bluck, Kenneth B. Ling, Katharine Ingram, Maria Ciaccia, Jamie A. Leitch, Duncan L. Browne

We write to correct the oxygen balance formulas that appear in Reference 5. Reference 5 with the correct formulas should appear as follows:

5. (a) Determination of oxygen balance - The oxygen balance provides a measure of the oxygen available within the molecule for complete combustion. This indicator can only be used if there are oxygen atoms in the molecule. The explosive power (energy release) of the material is considered to be a maximum risk at equivalence, i.e., zero oxygen balance. A deficiency of oxygen will give a negative balance, whereas an excess of oxygen will give a positive balance. The oxygen balance is determined for the following chemical reaction:
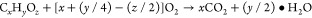
and can be calculated from the following equation:
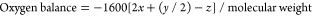
where *x*, *y* and *z* refer to the number of carbon, hydrogen and oxygen atoms, respectively, in the molecule. *Note: The molecular weight must include all atoms not just C, H and O.* Most explosives have oxygen balances between −100 and +40. However, any material with an oxygen balance more positive than −200 should be treated with caution.

(b) Recommendations on the Transport of Dangerous Goods Model Regulations - 22nd Revised Edition (Vol. I & II) ISBN: 9789211391886.

(c) Recommendations on the Transport of Dangerous Goods: Manual of Tests and Criteria - Sixth Revised Edition, ISBN: 9789211391626.

